# Effects of 5-azaC on Iridoid Glycoside Accumulation and DNA Methylation in *Rehmannia glutinosa*

**DOI:** 10.3389/fpls.2022.913717

**Published:** 2022-06-23

**Authors:** Tianyu Dong, Shanglin Song, Ying Wang, Ruixue Yang, Peilei Chen, Jiuchang Su, Xinru Ding, Yongkang Liu, Hongying Duan

**Affiliations:** ^1^College of Life Sciences, Henan Normal University, Xinxiang, China; ^2^Henan International Joint Laboratory of Aquatic Toxicology and Health Protection, College of Life Science, Henan Normal University, Xinxiang, China; ^3^Agricultural Research Institute of Wenxian County, Wenxian, China

**Keywords:** *Rehmannia glutinosa*, iridoid glycoside, DNA methylation, 5-azaC, RNA-Seq

## Abstract

Iridoid glycoside is the important secondary metabolite and the main active component in *Rehmannia glutinosa*. However, the mechanisms that underlie the regulation of iridoid glycoside biosynthesis remain poorly understood in *R. glutinosa*. Herein, the analysis of RNA-seq data revealed that 3,394 unigenes related to the biosynthesis of secondary metabolites were identified in *R. glutinosa*. A total of 357 unigenes were involved in iridoid glycoside synthesis, in which the highly conservative genes, such as *DXS, DXR, GPPS, G10H*, and *10HGO*, in organisms were overexpressed. The analysis of the above genes confirmed that the co-occurrence ratio of *DXS, DXR*, and *GPPS* was high in plants. Further, our results showed that under normal and 5-azacytidine (5-azaC) treatment, the expression levels of *DXS, DXR, GPPS, G10H*, and *10HGO* were consistent with the iridoid glycoside accumulation in *R. glutinosa*, in which the application of the different concentrations of 5-azaC, especially 50 μM 5-azaC, could significantly upregulate the expression of five genes above and iridoid glycoside content. In addition, the changes in the spatiotemporal specificity of degree and levels of DNA methylation were observed in *R. glutinosa*, in which the hemi-methylation was the main reason for the change in DNA methylation levels. Similar to the changes in 5-methyl cytosine (5mC) content, the DNA demethylation could be induced by 5-azaC and responded in a dose-dependent manner to 15, 50, and 100 μM 5-azaC. Taken together, the expression of iridoid glycoside synthesis gene was upregulated by the demethylation in *R. glutinosa*, followed by triggering the iridoid glycoside accumulation. These findings not only identify the key genes of iridoid glycoside synthesis from *R. glutinosa*, but also expand our current knowledge of the function of methylation in iridoid glycoside accumulation.

## Introduction

Plant secondary metabolites (SMs) not only play a pivotal role in plant growth and environmental adaptation, but also are used as medicines by humans because of their rich pharmacological effects (Li Y. et al., 2020). There are diverse SMs in plants, such as nitrogen-containing compounds, phenolic compounds, and terpenes (Fang et al., 2011). The abundant and widely distributed terpenes are synthesized from isoprene diphosphate (IPP) and dimethylallyl diphosphate (DMAPP) (Pu et al., 2021). Iridoid glycoside belongs to monoterpene, and its biosynthetic precursors IPP and DMAPP mainly come from 2-C-methyl-D-erythritol-4-phosphoric acid pathway (MEP) (Choi and Sano, 2007; Li et al., 2010; Liu et al., 2016). The study of iridoid glycoside biosynthesis is recently focused on a series of key enzymes in MEP pathway and terpene synthetase. 1-deoxyxylulose-5-phosphate synthetase (DXS) is the first rate-limiting enzyme in MEP pathway, which catalyzes the reaction of pyruvate and glycerol-3-phosphate to produce 1-deoxyxylulose-5-phosphate (DXP) (Sprenger et al., 1997). DXP is catalyzed to form MEP by 1-deoxy-D-xylulose-5-phosphate reductase (DXR) (Lichtenthaler, 1999; Carretero-Paulet, 2002). Geranyl diphosphate (GPP), the precursor of monoterpene, is synthesized from IPP and DMAPP under the catalysis of geranyl pyrophosphate synthase (GPPS). The production of GPP is the first step in the synthesis of monoterpene (Wang et al., 2013; Xi et al., 2016). Subsequently, the oxidation, reduction, glycosylation, and methylation reactions occur under the catalysis of various functional enzymes (Burlat et al., 2004; Geu-Flores et al., 2012; Wu and Liu, 2017). Geraniol 10-hydroxylase (G10H) is a key enzyme in monoterpene synthesis that can catalyze the oxidation of 10-hydroxygeraniol to 10-oxogeraniol (Zhao et al., 2007; Sung et al., 2011), and 10-oxogeraniol is further oxidized to 10-oxogeranial by 8-hydroxygeraniol dehydrogenase (10HGO) which is a NAPDH-dependent cytochrome P450 monoterpene oxidase (Oudin et al., 2007; Verma et al., 2012) (Supplementary Figure 1).

It is well known that plant secondary metabolism is a quite complex physiological process and is dramatically affected at transcription level (Borges et al., 2017; Kurepin et al., 2017; Sanchita and Sharma, 2018). As a common epigenetic modification, DNA methylation is closely related to gene expression, transposon silencing, and heterochromatin formation (Qian et al., 2014; Bharti et al., 2015; Wang et al., 2021). It is a crucial means to regulate growth and development or responses to environments (Yamamuro et al., 2014; Zhou S. et al., 2016; Duan et al., 2018). The level and status of DNA methylation are associated with plant growth and development, environmental changes, and plant species (Candaele et al., 2014; Duan et al., 2018). The abnormal state of DNA methylation can lead to the aberrant growth and development of plant (Yamamuro et al., 2014). Many studies have found that DNA methylation is involved in plant secondary metabolism and affects SMs accumulation (Tan et al., 2017; Wang et al., 2019; Badad et al., 2021). Under the treatment of DNA methylation inhibitor 5-azaC, the content of polysaccharides and alkaloids was increased significantly in *Dendrobium* (Ni et al., 2014), and the biosynthesis of tanshinones was enhanced in *Salvia miltiorrhiza* hairy roots (Jiang et al., 2018; Li et al., 2018). The accumulation of ganoderic acid and the upregulated expression of related synthetase enzyme genes were also induced by the applied 5-azaC in *Ganoderma lucidum* (Lan, 2016). Although the relationship between DNA methylation and SMs accumulation in plants has been explored, the mechanism of DNA methylation regulating plant secondary metabolism is still unclear.

*Rehmannia glutinosa*, a perennial herb with great medicinal and economic values, belongs to Rehmannia genus of Scrophulariaceae (Chen et al., 2021). Iridoid glycoside is one of the main active components in *R. glutinosa* (Wang et al., 2019). However, the regulatory mechanism of iridoid glycoside biosynthesis has not been reported in *R. glutinosa*. There are few studies on the relationship between DNA methylation and iridoid glycoside accumulation in plants. To reveal the role of DNA methylation modification in iridoid glycoside synthesis in *R. glutinosa*, the iridoid glycoside synthetase genes were cloned and their expression, iridoid glycoside accumulation, and DNA methylation in *R. glutinosa* were analyzed. The results not only provide a scientific basis for development and utilization of *R. glutinosa*, but also establish a foundation for the studies on epigenetic mechanisms underlying plant secondary metabolism.

## Materials and Methods

### Treatment and Cultivation of *R. glutinosa*

In this experiment, *Rehmannia glutinosa* “Jinjiu” was used. In early April, root tubers were planted in test field of Henan Normal University, Xinxiang City, Henan, China (N35°18′13.71″, E113°55′15.05″).

Roots and leaves of *R. glutinosa* were collected, respectively, at Stage E (the elongate stage: the root is fleshy and cylindrical in early June), Stage I (the intumescent stage: the root displays expansion in the middle of September), and Stage M (the mature stage: the root is spindle-shaped in early December) and then stored at −80°C. In addition, 100 collected root tubers of *R. glutinosa* in each stage were used for the treatment of 5-azacytidine (5-azaC) with three replicates. In the pilot experiment, *R. glutinosa* seedlings were treated with 15, 50, 100, 150, and 250 μM 5-azaC. According to the effects of 5-azaC on the growth of *R. glutinosa* seedlings, 15, 50, and 100 μM 5-azaC were selected further experiments. After sterilized for 10 min by 0.1% HgCl_2_, root tubers of *R. glutinosa* were washed for 3–5 min with sterile water, and then, these root tubers were treated with 15, 50, and 100 μM 5-azaC, respectively.

### RNA-Seq Data Analysis

The total RNA in Jinjiu root was extracted using a miRNA Isolation Kit (Ambion). mRNA of each sample was isolated from the total RNA by using beads with oligo (dT) and then was added with a fragmentation buffer to cleave the mRNA into short fragments. The mRNA above was employed as templates for the synthesis of first-strand cDNA using random hexamer primers. Sequencing of cDNA library was performed by Illumina NovaSeq technology. After obtaining the raw sequencing data, the adapter contaminations and trimming nucleotides with low-quality score were removed. After getting high-quality sequencing data by Trinity, reads are fragmented into smaller pieces, known as K-mer (Grabherr et al., 2011). These K-mers are then used as seeds to be extended into contigs and then component basing on contig overlapping. Finally, De Bruijn was applied to recognize transcripts in the components. Then, the randomness of mRNA fragments and the integrity of mRNA were checked by examining the distribution of inserts on unigenes, and the dispersion of inserts was evaluated by counting the length of inserts. The sufficiency of mapped data was checked by generating saturation curve of total gene number and total reads number.

The functions of the unigenes were annotated as NCBI non-redundant protein (NR), Swiss-Prot, COG, KOG, eggNOG 4.5, and KEGG (Buchfink et al., 2015). The KEGG Orthology of unigenes was analyzed by KOBAS (Xie et al., 2011), and GO Orthology of unigenes was analyzed by InterProScan (Jones et al., 2014). After predicting the amino acid sequence of unigenes, the domain was annotated by HMMER and Pfam database.

### The Extraction of Nucleic Acid

Genomic DNA was extracted from root and leaf in *R. glutinosa* (Duan et al., 2018). The precipitated DNA was washed twice with 70% ethanol solution, and the dried DNA was dissolved in double-distilled water; then, 1% RNase was added to DNA solution. The integrity of genomic DNA was detected by 0.8% agarose gel electrophoresis, and the yield and purity of genomic DNA were determined by spectrophotometry.

Total RNA extraction from root and leaf in *R. glutinosa* was performed as described by Chen et al. (2021). DNase treatment and phenol–chloroform extraction were used to remove DNA. The integrity of total RNA was detected by 1.0% agarose gel. The yield and purity of total RNA were estimated. In addition, the first-strand cDNA was synthesized according to the instruction of TaKaRa kit (TaKaRa, Japan).

### Cloning and Analysis of Target Genes

The synthetase genes of iridoid glycoside of *R. glutinosa* were cloned, such as *DXS, DXR, GPPS, G10H*, and *10HGO*. The full-length cDNA sequences were obtained by electronic cloning technology as follows: The partial sequences of target genes from transcriptome sequencing were used as probes to search SRA database by BLASTN. SRA sequence of *R. glutinosa* that matched the probe sequence was compared, spliced, and extended by DNAMAN 7.0, which was continually carried out until no more SRA sequence can be obtained.

According to cDNA sequence obtained by electronic cloning, the corresponding primers were designed. PCR product was detected by agarose gel electrophoresis. Next, the target fragments were cut and sequenced.

The following methods were adopted to analyze target genes: ORF of genes was predicted by ORF finder program (http://www.ncbi.nlm.nih.gov/gorf/gorf.html); the amino acid sequence, molecular weight (MW), isoelectric point (PI), and other physicochemical properties were predicted by ExPASy proteomics server (http://www.expasy.ch/tools/protparam.html); the subcellular localization was predicted by Plant-mPLoc (http://www.csbio.sjtu.edu.cn/bioinf/plant-multi/). In addition, the conservative analysis of *DXS, DXR, GPPS, G10H*, or *10HGO* was performed by NCBI CD-search and NCBI BLASTP (https://blast.ncbi.nlm.nih.gov/Blast.cgi). Amino acid sequences were compared, and phylogenetic trees were constructed by MEGA 7.0. Based on STRING, the co-occurrence of *DXS, DXR, GPPS, G10H*, and *10HGO* was analyzed (https://string-db.org/).

### Quantitative Real-Time PCR

The quantitative real-time PCR (qRT-PCR) was carried out in LightCycler 96 fluorescence quantitative PCR instrument. *TIP41* was used as the internal reference gene, and all the primer sequences were presented in Supplementary Table 1. According to AceQ Qpcr SYBR Green MasterMix kit (Vazyme, Nanjing, China) instructions, the configuration of reaction system is performed. The expression level of target gene was calculated by 2^−Δ*ΔCt*^ (Wang et al., 2021).

### Determination of Iridoid Glycoside

The fresh roots and leaves from *R. glutinosa* were dehydrated in the oven at 50°C to constant weight. The dried samples were then ground into powder. One gram powder was mixed with 10 mL 70% ethanol solution in 100 mL plugged conical bottle, and the mixture was filtered by ultrasonic technique. The filtrate was filtered and shaken in a 50 mL volumetric flask. Then, 2.5 mL filtrate was diluted to 50 mL.

In this experiment, catalpol was used as reference substance. The determination of iridoid glycoside was conducted as follows: 1 mL of test solution and 2 mL of 1 M HCL were mixed in 10 mL plugged test tube. The tube was heated in a water bath at 90°C for 15 min and then placed at room temperature for 15 min. The mixture was added with 0.5 mL of dinitrophenylhydrazine ethanol solution and 30 mL 70% ethanol solution. Then, this reaction solution was placed at room temperature for 1 h and determined by spectrophotometer at 463 nm.

### Determination of 5-Methyl Cytosine

The 5-methyl cytosine (5mC) content in *R. glutinosa* was detected by high-performance liquid chromatography (HPLC; Li J. Y. et al., 2020). After genomic DNA was hydrolyzed in order with DNase I, nuclease P1, and alkaline phosphatase, this digestion system was centrifuged for 5 min at 12,000 rpm. The supernatant was filtered with 0.45 μM organic microfiltration membrane, and 5mC content was determined by HPLC.

These chromatography conditions performed in this study were as follows: The mobile phase was composed of 50 mM KH_2_PO_4_ and 8% methanol (92:8) with 0.5 ml/min flow velocity, and the analytical column was Agilent C18 Zorbax XDB column (4.6 × 150 mm, 5 μm particle size). Furthermore, non-methylated cytosines (C) and 5mC in genomic DNA could be detected at 285 nm according to the retention time of C and 5mC.

### MSAP Amplification

The methylation-sensitive amplified polymorphism (MSAP) amplification was performed as described by Duan et al. (2020). Genomic DNA was digested with EcoRI/Msp? (M) and EcoRI/HpaII (H) and then was ligated with EcoRI adapter and MspI-HpaII adapter for 15 h at 16°C. Subsequently, the products above were prepared for MSAP amplification. After the MSAP pre-amplification, the product was used as template in MSAP selective amplification.

DNA methylation levels were quantified by MSAP binary data (Duan et al., 2018). The presence or absence of one clear band was, respectively, scored as “1” and “0.” On the basis of the presence or absence in H and M, the banding patterns of DNA methylation were divided into three classes (Supplementary Figure 2), in which (1) the presence of band in H and M was considered to be no-methylation (class I), (2) the presence of band only in H was considered to be DNA hemi-methylation (class II), and (3) the presence of band only in M was considered to be DNA full methylation (class III). In addition, DNA total methylation level (%) = (II + III)/(I + II + III) ×100, DNA hemi-methylation level (%) = II/(I + II + III) ×100, DNA full methylation level (%) = III/(I + II + III) ×100.

### Statistical Analysis

All data were analyzed using the SPSS 17.0 software (SPSS Inc, Chicago, USA) and presented as the means ± SD from three independent experiments in triplicate for each. The statistical analysis of all data was performed by one-way analysis of variance followed by Duncan's multiple comparison test.

## Results

### Identification and Annotation of Unigenes Related to Iridoid Glycoside Biosynthesis

**Figure 1:**
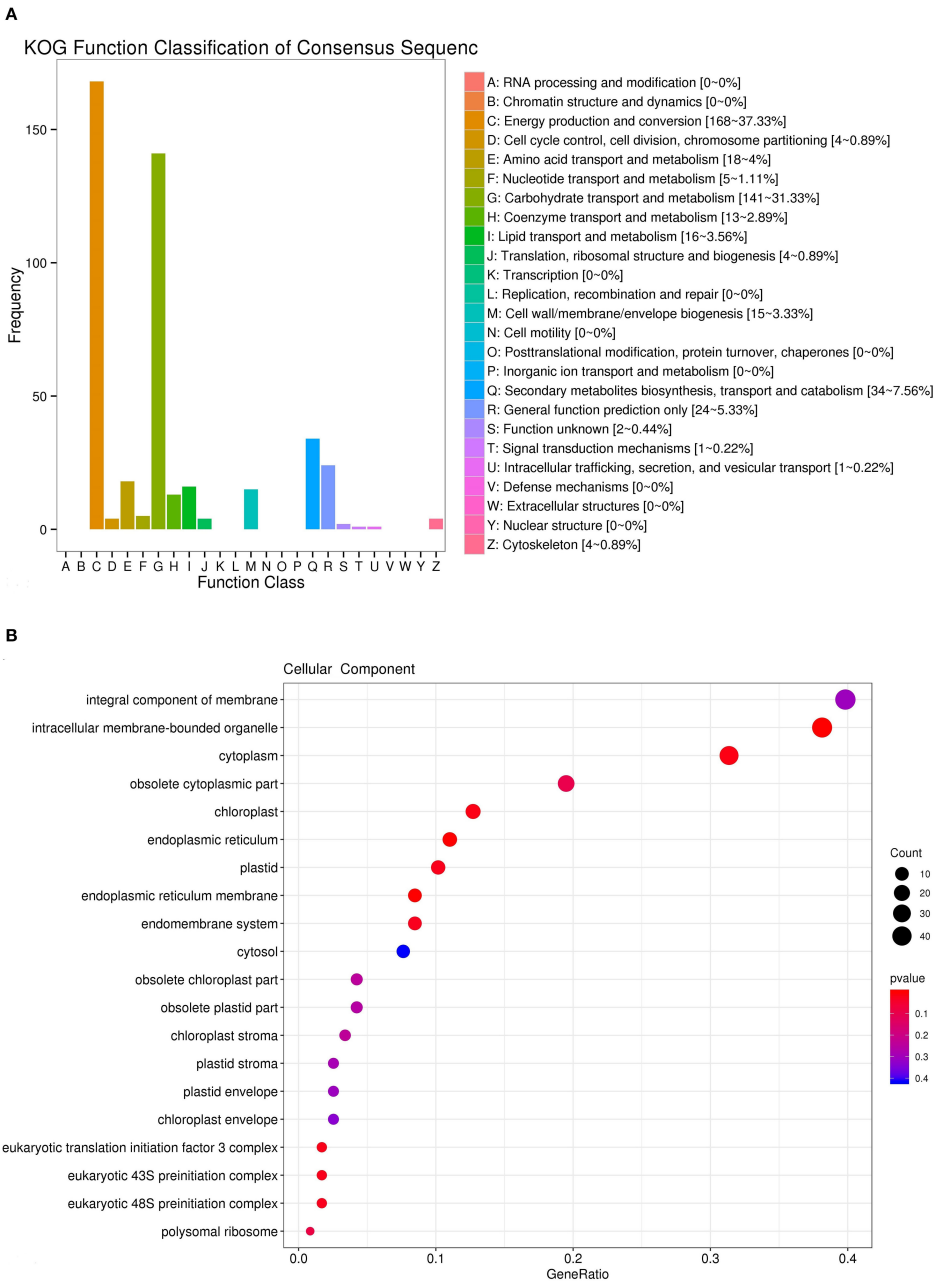
Identification and annotation of unigenes involved in iridoid glycoside of *R. glutinosa*. **(A)** KOG function classification of unigenes. **(B)** Cellular component enrichment of unigenes.

To identify the genes of iridoid glycoside biosynthesis, RNA-sequencing (RNA-seq) technology was applied to analyze the transcriptomic data from *R. glutinosa* (Jinjiu). For the RNA-seq analysis, the adaptor and low quality of raw data were removed. All clean reads were assembled into expressed sequence tag clusters (contigs), and the above contigs were *de novo* assembled into transcripts using the Trinity in paired-end method, in which yielded a total of 75,698 unigenes. The analysis of KEGG pathway showed that 3,394, 328, and 357 unigenes were involved in the secondary metabolite biosynthesis, terpenoid biosynthesis, and iridoid glycoside biosynthesis, respectively (Supplementary Tables 2–4). A total of 357 unigenes of iridoid glycoside biosynthesis were further annotated by using KOG database, in which 37.33, 31.33, and 7.65% unigenes were clustered to the energy production and conversion, the carbohydrate transport and metabolism, and the secondary metabolites biosynthesis, transport, and catabolism, respectively (Figure 1A). The cellular component enrichment from GO database confirmed that the above 357 unigenes were located in the integral component of membrane, intracellular membrane-bounded organelle, cytoplasm, obsolete cytoplasmic part, chloroplast, endoplasmic reticulum, plastid, endoplasmic reticulum membrane, etc. (Figure 1B).

**Figure 2:**
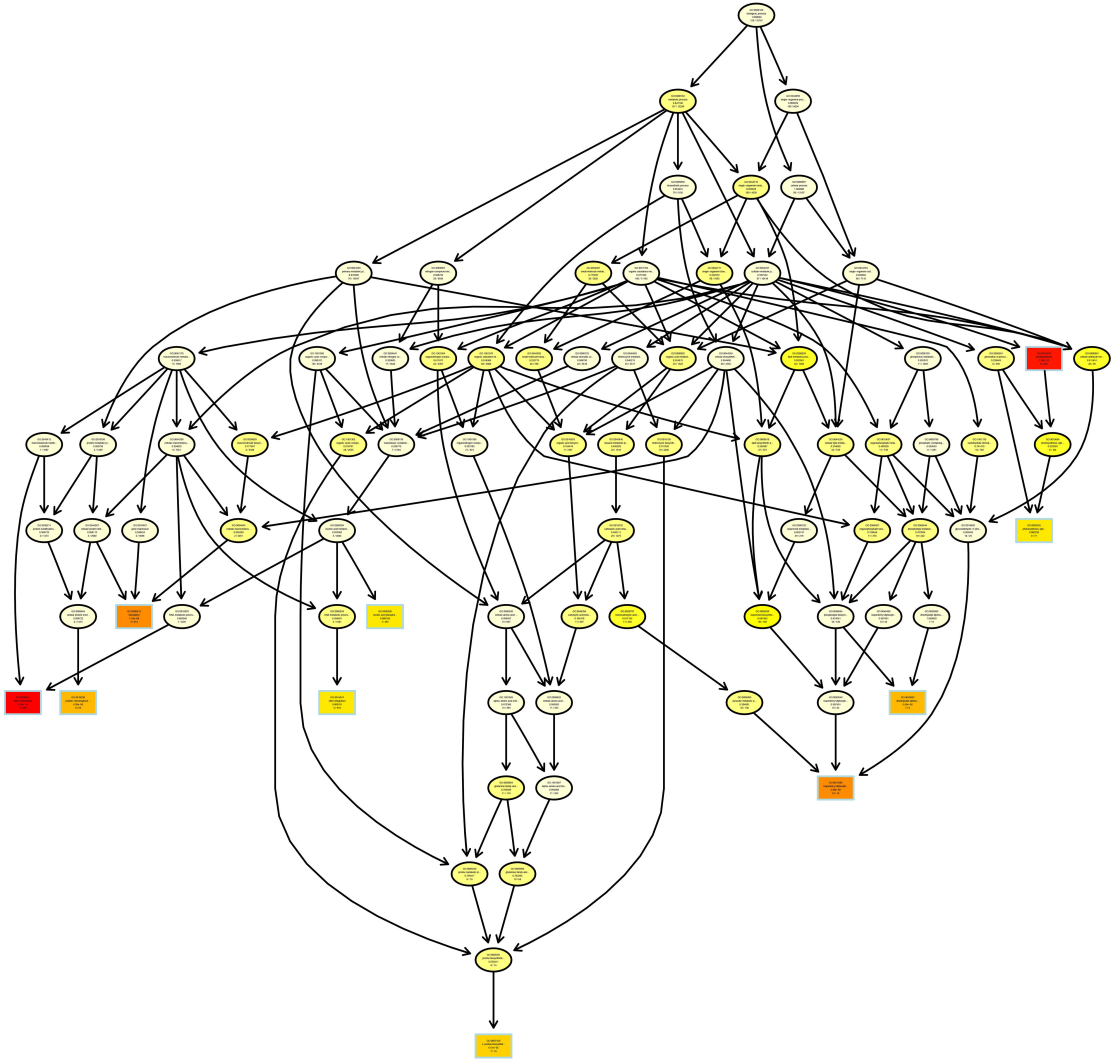
TopGO directed acyclic graph of unigenes related to iridoid glycoside synthesis. Boxes represent top10 GO term with the most significant enrichment. Each box (or ellipse) contains a description and a *P*-value. Arrow represents containment relationship.

Subsequently, topGO lineage was plotted, and the significance of each node was analyzed. A total of 82 GO terms were classified into 13 gradations (Figure 2). A total of seven unigenes were significantly enriched in dimethylallyl diphosphate biosynthetic process; 10 unigenes were obviously gathered in isopentenyl diphosphate biosynthetic process and methylerythritol 4-phosphate pathway; and seven unigenes were involved in L-proline biosynthetic process.

### Cloning and Analysis of Iridoid Glycoside Synthetase Genes

To further explore the key genes of iridoid glycoside biosynthesis, five genes, such as *DXS, DXR, GPPS, G10H*, and *10HGO*, were cloned from *R. glutinosa* (Supplementary Figure 3). This gene structure was analyzed (Supplementary Figures 4, 5, Supplementary Table 5). The physicochemical properties of amino acids were predicted. DXS, GPPS, and G10H were hydrophilic protein, and DXR and 10HGO were hydrophobic protein. 10HGO was located in cytoplasm, while others were located in chloroplastb (Supplementary Table 6). The phylogenetic trees showed that *DXS, GPPS, G10H*, and *10HGO* from *R. glutinosa* could be clustered into one branch with *Scutellaria barbata, Sesamum indicum, Handroanthus impetiginosus*, and *Striga asiatica*, respectively (Supplementary Figure 6). The homologous genes of *DXS, DXR, GPPS, G10H*, and *10HGO* in plants were searched by BLAST (Supplementary Figure 7, Supplementary Table 7). The homology of *DXS, GPPS, G10H*, and *10HGO* was high with those in *Sesamum indicum*, and *DXR* showed high homology with *Handroanthus impetiginosus* or *Osmanthus fragrans*.

**Figure 3:**
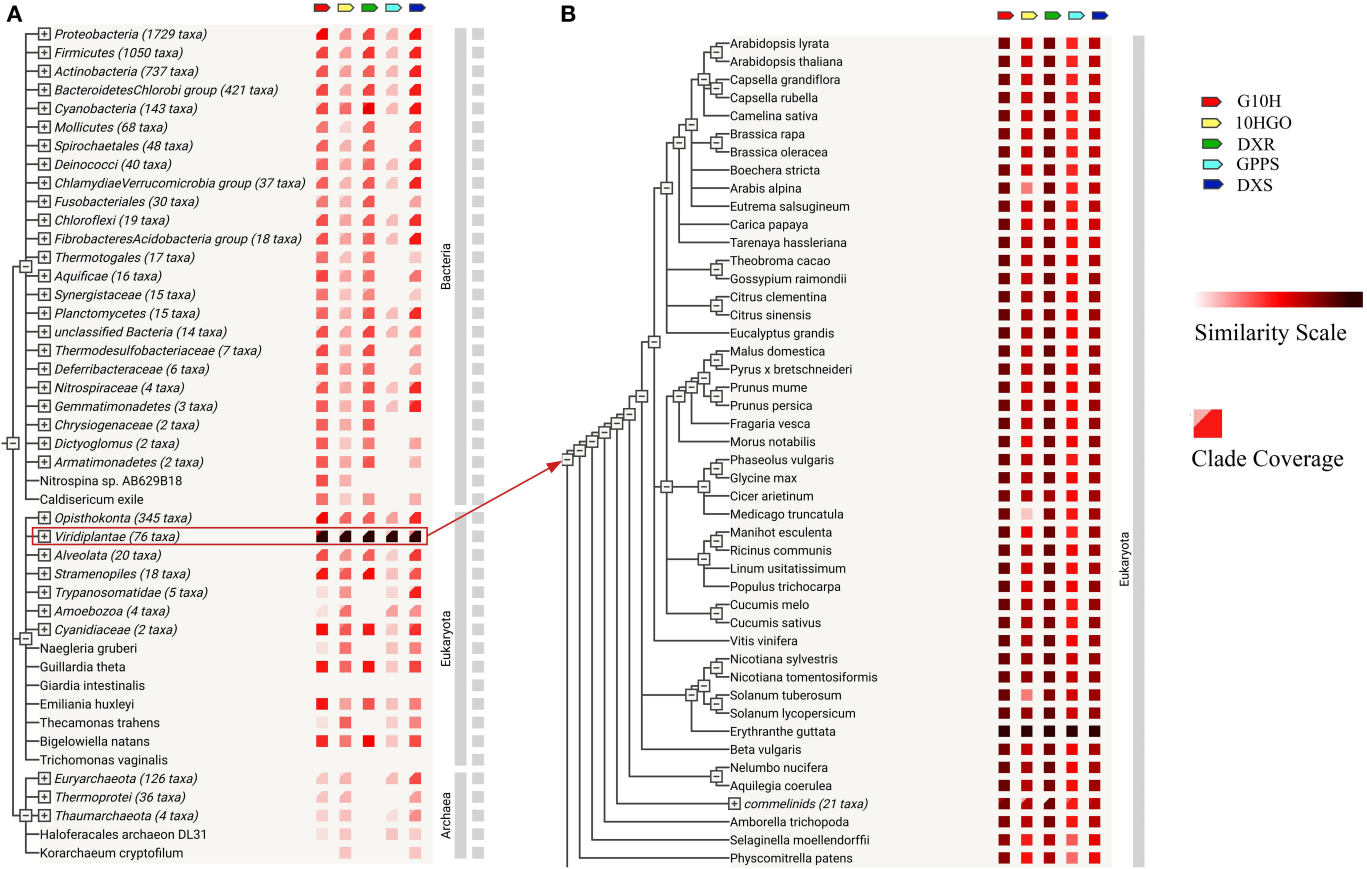
Co-occurrence on iridoid glycoside synthetase genes of *R. glutinosa*. **(A)** Gene co-occurrence of *DXS, DXR, GPPS, G10H*, and *10HGO* in organism. **(B)** The co-occurrence of *DXS, DXR, GPPS, G10H*, and *10HGO* in plants. Similarity scale: The color indicates the similarity of gene and its homology in the given STRING. Clade coverage: Two distinct colors, respectively, denote the lowest and highest similarity observed within the clade.

Based on STRING database, the gene co-occurrence on *DXS, DXR, GPPS, G10H*, and *10HGO* of *R. glutinosa* was analyzed (Figure 3), suggesting that they were very conservative in evolution and exist in Bacteria, Eukaryota, and Archaea (Figure 3A). Further analysis confirmed that *DXS, DXR, GPPS, G10H*, and *10HGO* of *R. glutinosa* showed high similarity with their homologs in Viridiplantae (Figure 3B), especially *Erythranthe guttata, Aquilegia coerulea, Citrus clementina, Citrus sinensis, Gossypium raimondii, Eucalyptus grandis, Malus domestica, Morus notabilis*, and *Nelumbo nucifera* (Supplementary Table 8). As shown in Table 1, *DXS, DXR, GPPS, G10H*, and *10HGO* were matched, respectively, by many genes in Viridiplantae, whose gene co-occurrence was found in 19 species in plant kingdom, such as *Aquilegia coerulea, Citrus clementine, Citrus sinensis, Erythranthe guttata, Eucalyptus grandis*, etc. *DXS, DXR, GPPS*, and *10HGO* were matched in seven species in plants, respectively, in which less co-occurrence was observed in *G10H. DXS, DXR*, and *GPPS* of *R. glutinosa* were matched, respectively, in 33 species, such as *Aegilops tauschii, Amborella trichopoda, Boechera stricta, Brassica rapa, Camelina sativa*, and *Zea mays*. In addition, the co-occurrence of *DXS, DXR*, or *GPPS* in plants was higher (90% or so), and the co-occurrence of *G10H* and *10HGO* in plants was only 30.6 and 38.9%, respectively (Table 1).

**Table 1:** Co-occurrence on DXS, DXR, GPPS, G10H, or 10HGO family.

**No. of queries matched**	**Plant organism**	**Gene co-occurrence**				
		***DXS***	***DXR***	***10HGO***	***G10H***	***GPPS***
5	*Aquilegia coerulea, Citrus clementine, Citrus sinensis Erythranthe guttata, Eucalyptus grandis, Gossypium raimondii, Malus domestica, Morus notabilis, Nelumbo nucifera, Nicotiana sylvestris, Nicotiana tomentosiformis, Populus trichocarpa, Prunus mume, Prunus persica, Pyrus × bretschneideri, Ricinus communis, Solanum lycopersicum, Theobroma cacao, Vitis vinifera*	✓	✓	✓	✓	✓
4	*Cicer arietinum, Cucumis sativus, Fragaria vesca, Glycine max, Linum usitatissimum, Manihot esculenta, Phaseolus vulgaris*	✓	✓	✓		✓
	*Beta vulgaris*	✓	✓		✓	✓
	*Solanum tuberosum*	✓	✓	✓	✓	
3	*Aegilops tauschii, Amborella trichopoda, Arabidopsis lyrata, Arabidopsis thaliana, Boechera stricta, Brachypodium distachyon, Brassica oleracea, Brassica rapa, Camelina sativa, Capsella grandiflora, Capsella rubella, Carica papaya, Cucumis melo, Eutrema salsugineum, Hordeum vulgare, Leersia perrieri, Musa acuminata, Oryza barthii, Oryza brachyantha, Oryza glaberrima, Oryza glumipatula, Oryza nivara, Oryza punctata, Oryza rufipogon, Oryza sativa, Panicum virgatum, Phoenix dactylifera, Physcomitrella patens, Selaginella moellendorffii, Setaria italic, Sorghum bicolor, Tarenaya hassleriana, Zea mays*	✓	✓			✓
	*Medicago truncatula*	✓	✓	✓		
2	*Arabis alpina*	✓	✓			
	*Triticum aestivum*		✓			✓
1	*Chlorella variabilis, Coccomyxa subellipsoidea, Triticum urartu, Volvox carteri*		✓			
	*Micromonas pusilla, Oryza meridionalis, Ostreococcus lucimarinus, Ostreococcus tauri*					✓
Rate of gene co-occurrence (%)		87.5	94.4	38.9	30.6	90.3

*The symbol ✓ indicates the presence of relative homologous gene for DXS, DXR, 10HGO, G10H, or GPPS of R. glutinosa*.

### Expression Pattern of *DXS, DXR, GPPS, G10H*, and *10HGO*

**Figure 4:**
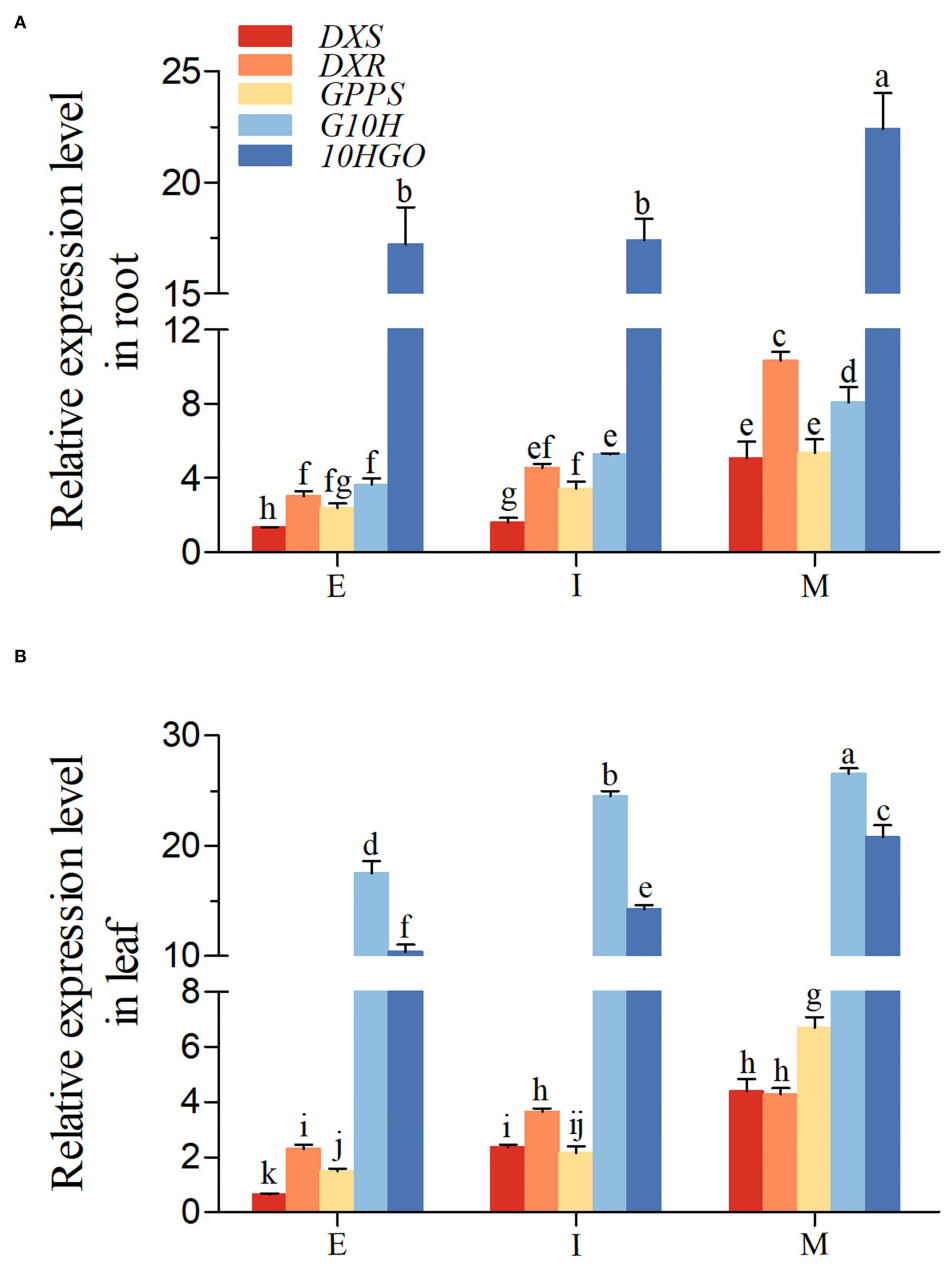
Expression of iridoid glycoside synthetase genes in *R. glutinosa*. **(A,B)** respectively, represent the expression of *DXS, DXR, GPPS, G10H*, and *10HGO* in Jinjiu root and Jinjiu leaf of *R. glutinosa*. E, I, and M represent elongation stage, intumescent stage, or maturation stage of *R. glutinosa*, separately. The error bar is standard error of mean, and the lower letter above the bar indicates the significant difference among different growth stages of *R. glutinosa* (*P* < 0.05).

Next, the expression of *DXS, DXR, GPPS, G10H*, and *10HGO* was gain insighted in *R. glutinosa*. As shown in Figures 4A,B, the expression level of five genes above in root and leaf was increased gradually from E to M stage. The expression level of *DXR* and *10HGO*, especially *10HGO*, in root was higher than those in leaf at E, I, and M stage, but the opposite trends were observed in *G10H*. Unlike the I stage, our results revealed that at E and M stage, the expression level of *DXS* in root was higher than those in leaf. Although the *GPPS* transcript level in root was higher than those in leaf at E and I stage, the *GPPS* expression in root was inhibited at M stage.

### Effects of 5-azaC Treatment on Accumulation of Iridoid Glycoside

**Figure 5:**
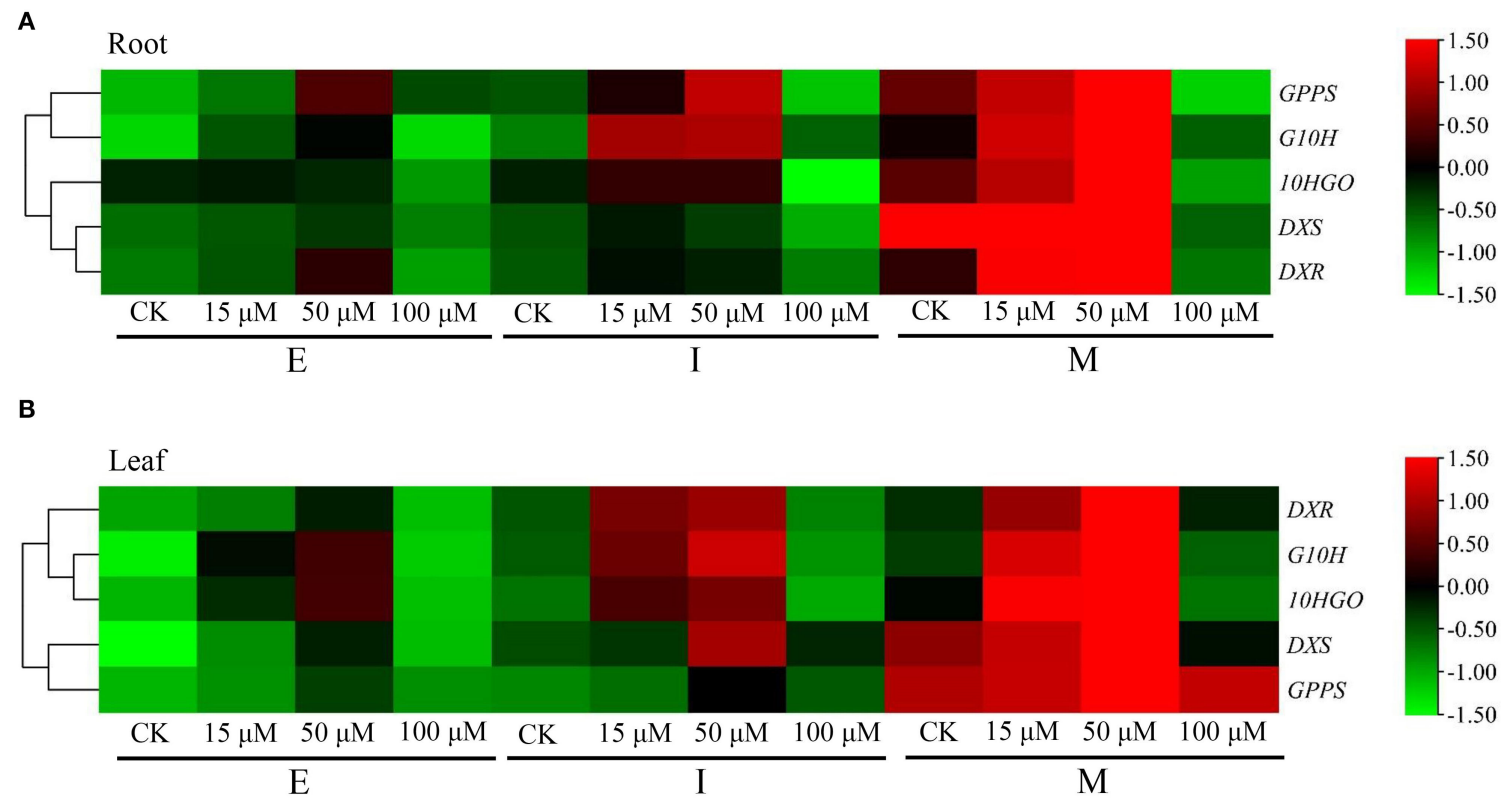
Expression pattern of iridoid glycoside synthetase genes in *R. glutinosa* treated with 5-azaC. **(A,B)** respectively, represent the relative expression level of *DXS, DXR, GPPS, G10H*, and *10HGO* in Jinjiu root and Jinjiu leaf of *R. glutinosa* treated with 5-azaC, respectively.

To explore the effects of DNA methylation on the gene expression of *DXS, DXR, GPPS, G10H*, and *10HGO*, the 15, 50, and 100 μM 5-azaC, a DNA methylation inhibitor, were applicated in *R. glutinosa* at E, I, and M stage, respectively. Unexpectedly, our results showed that at E, I, and M stage, the expressions of *DXS, DXR, GPPS, G10H*, and *10HGO* in root and leaf were upregulated under 15 or 50 μM 5-azaC treatment, compared with the control groups (Figures 5A,B). However, the expression of *DXS, DXR, GPPS, G10H*, and *10HGO* in root was downregulated at M stage under 100 μM 5-azaC treatment (Figure 5A), but the similar trends were not observed in leaf. In addition, the expression level of these genes in both root and leaf reached the highest at M stage under 50 μM 5-azaC treatment (Figures 5A,B). The classification analysis showed that in root, the expression trend between *DXS* and *DXR* was similar, and the expression of *GPPS* and *G10H* revealed the similar trend (Figure 5A). Unlike, *10HGO* was divided into one branch alone in root. In leaf, *DXS* showed the expression trend similar to those of *GPPS*, and the expression trend of *10HGO* was similar to those of *G10H* (Figure 5B). However, *DXR* was divided into one branch (Figure 5B).

**Figure 6:**
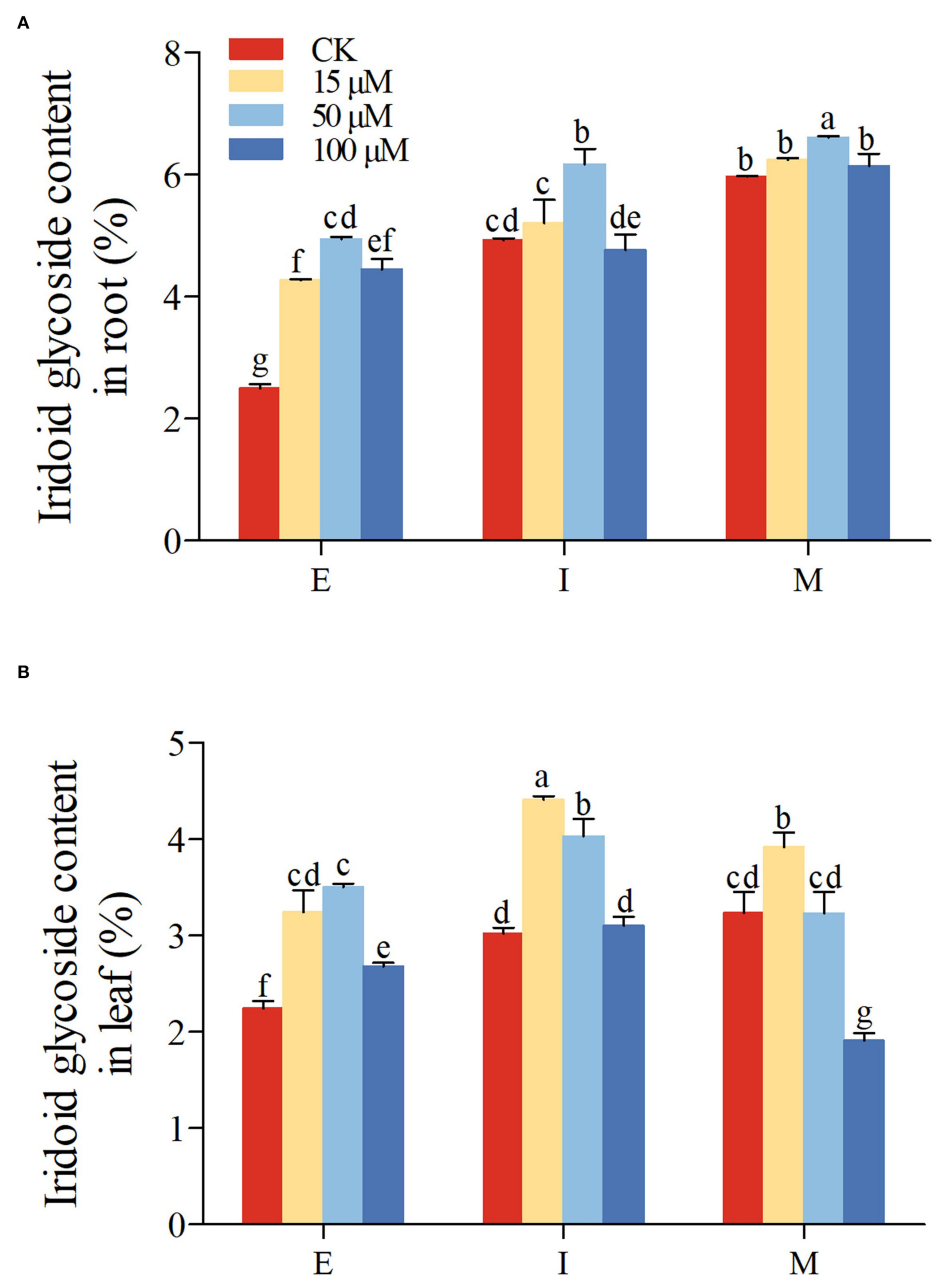
Effect of 5-azaC on iridoid glycoside accumulation in *R. glutinosa*. **(A,B)** respectively, represent the content of iridoid glycoside in Jinjiu root and Jinjiu leaf of *R. glutinosa*, and E, I, and M represent elongation stage, intumescent stage, or maturation stage of *R. glutinosa*, separately. The error bar is standard error of mean, and the lower letter above the bar indicates the significant difference in root and leaf among different growth stages of *R. glutinosa* (*P* < 0.05).

Subsequently, the iridoid glycoside accumulation in root and leaf was further determined under normal and 5-azaC treatment. In control group, the iridoid glycoside accumulation in *R. glutinosa* was increased gradually from E to M stage in root (*P* < 0.05; Figure 6A). Although there were no significant differences of iridoid glycoside content in leaf between I and M stage, iridoid glycoside content of at both I and M stage was higher than those at E stage (Figure 6B). From E to M stage, the iridoid glycoside content in root (Figure 6A) was significantly higher than those in leaf (Figure 6B), especially M stage (nearly 2-fold). Compared with the control group, the iridoid glycoside content in root was significantly higher under 50 μM 5-azaC treatment at E, I, and M stage, especially E stage (Figure 6A). Unlike the I and M stage, both 15 and 100 μM 5-azaC treatment induced the iridoid glycoside accumulation in root at E stage, compared to control groups (Figure 6A). Compared with control, the application of 15 μM 5-azaC could induce the accumulation of iridoid glycoside in leaf at I stage (Figure 6B). Under 50 μM 5-azaC treatment, the iridoid glycoside content in root was higher than those in leaf (nearly 2-fold) at M stage (Figure 6B). Under 100 μM 5-azaC treatment, the iridoid glycoside content in root was above 3-fold than those in leaf at M stage (Figure 6B).

### Determination of Genomic DNA Methylation

**Figure 7:**
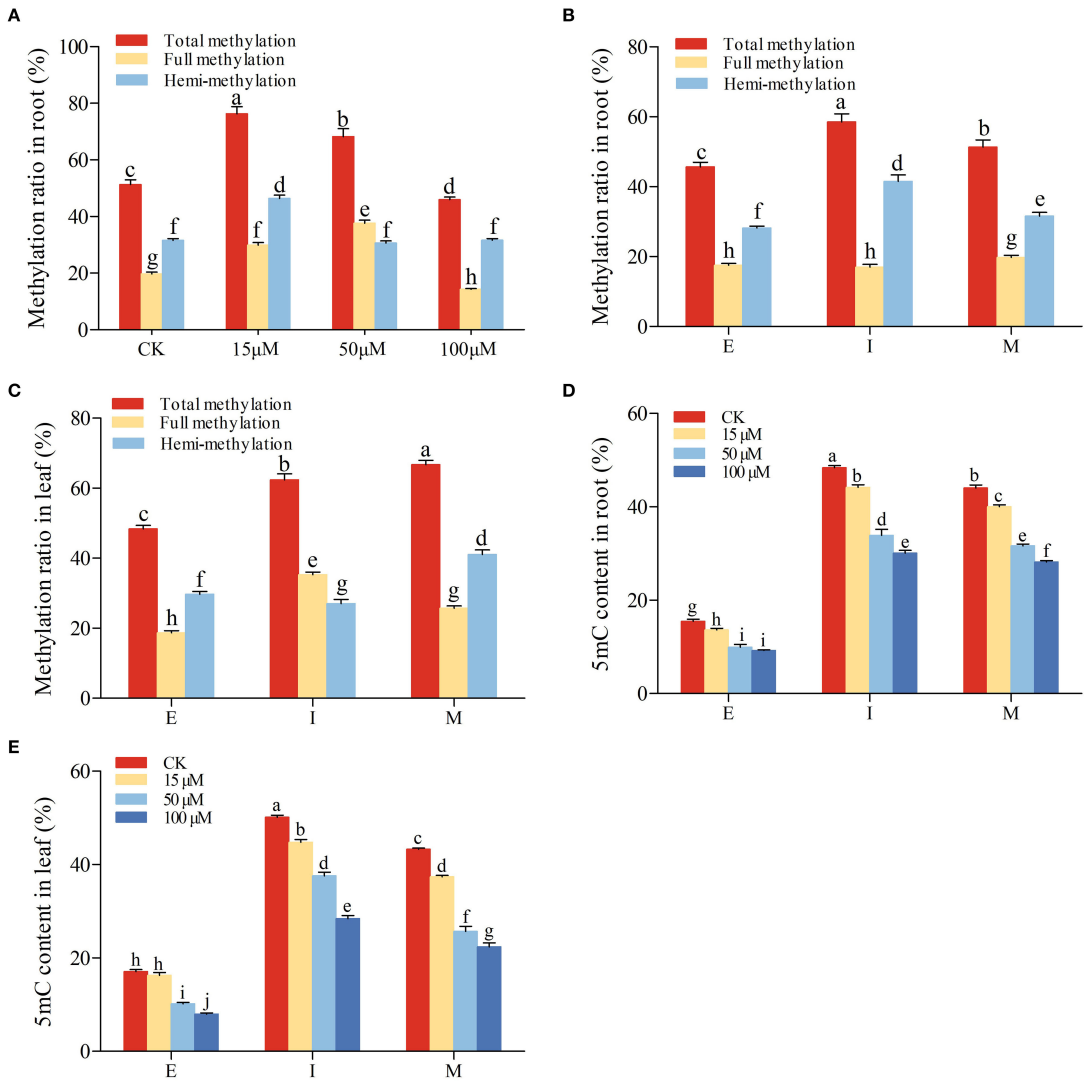
Genomic DNA methylation in *R. glutinosa*. **(A)** Methylation ratio in root at M stage under 5-azaC treatment. **(B,C)** represent methylation ratio in root or leaf of *R. glutinosa*; **(D,E)** represent 5mC content in root or leaf of *R. glutinosa* under 5-azaC treatment. E, I, and M represent elongation stage, intumescent stage, or maturation stage of *R. glutinosa*, respectively. The error bar is standard error of mean, and the lower letter above the bar indicates the significant difference (*P* < 0.05).

The above results have found that under 5-azaC treatment, the iridoid glycoside accumulation in root was higher than those in leaf at M stage. In order to explore the variation of genome methylation, the methylation ratio was detected by MSAP amplification in *R. glutinosa* at M stage under 5-azaC treatment (Figure 7A). The total and full methylation ratios in 15 μM and 50 μM 5-azaC treatment were higher than those in other groups. The hemi-methylation ratio reached the highest under 15 μM 5-azaC treatment, and the difference among other groups is very little. In addition, the total and full methylation ratios were decreased significantly under 100 μM 5-azaC treatment, compared with control group (Figure 7A).

To elucidate the changes in genomic DNA methylation, the full and hemi-methylation levels in both root and leaf were determined from E to M stage. According to full and hemi-methylation levels, the total methylation levels were calculated. Compared with leaf, the total and full methylation levels of root were lower at I and M stage (Figures 7B,C). As shown in Figure 7B, the total methylation and hemi-methylation levels presented increasing trend and reached the highest at I stage in root, while full methylation level was almost no change. Hemi-methylation levels in root were significantly higher than full methylation levels (Figure 7B), indicating that most CCGG sequences are keeping the state of hemi-methylation. Total methylation levels were also increased in leaf, especially M stage (*P* < 0.05) (Figure 7C).

Compared with the control, the 5mC accumulation in root and leaf was inhibited by the application of 5-azaC at E, I, and M stage (Figures 7D,E). Under the 5-azaC treatment, the 5mC contents in root and leaf were decreased gradually in dose-dependent manner (Figures 7D,E).

### Demethylation Occurs Upon the 5-azaC Treatment

To explore the dynamic changes in methylation sites under 5-azaC treatment, the methylation status of genomic DNA could be divided into two categories (B and C), in which the eight band types (B1–B4 and C1–C4) were identified, respectively (Table 2). The band patterns for the increase in DNA methylation were presented in type B, and it included the four categories: I → III, I → IV, II → III, and II → IV. By contrast, the band patterns for DNA demethylation were revealed by type C, in which four type C were defined as the IV → II, IV → I, III → II, and III → I. These results of Table 2 confirmed that under 15, 50, and 100 μM 5-azaC treatment, the band numbers of methylation in root were raised by 3, 7, and 5, respectively, compared to the control. As listed in Table 2, our results showed that compared to the control, the band numbers of demethylation in root under 15, 50, and 100 μM 5-azaC treatment were increased as 7, 20, and 25, respectively. Similar to the changes in methylation levels in root, our results further revealed that compared to control, the band numbers of methylation in leaf under 15, 50, and 100 μM 5-azaC treatment were increased as 4, 9, and 5, respectively (Table 2). Meanwhile, the band numbers of demethylation were increased as 9, 21, and 29, respectively (Table 2). However, the total band numbers of methylation and demethylation in leaf under 5-azaC treatment were more than those in root, especially under 100 μM 5-azaC treatment, suggesting that compared with root, the dynamic transformation of methylation was sensitive to 5-azaC treatment in leaf.

**Table 2:** Change in methylation pattern in *R. glutinosa* under 5-azaC treatment.

**Class**	**Band type**				**No. of sites**					
	**CK**		**Treat**		**Root**			**Leaf**		
	**H**	**M**	**H**	**M**	**CK-15μM**	**CK-50μM**	**CK-100μM**	**CK-15μM**	**CK-50μM**	**CK-100μM**
B1	1	1	0	1	0	1	1	0	2	2
B2	1	0	0	1	1	0	0	1	0	0
B3	1	0	0	0	2	6	4	3	7	3
B4	1	1	0	0	0	0	0	0	0	0
C1	0	1	1	1	3	6	8	4	8	9
C2	0	1	1	0	0	0	1	0	0	1
C3	0	0	1	0	4	12	9	5	10	13
C4	0	0	1	1	0	2	7	0	3	6

*B1–C4 represent change in methylation pattern in root or leaf of R. glutinosa under 5-azaC treatment as compared to the control group (CK), B1: I **→** III, B2: II **→** III, B3: II **→** IV, B4: I **→** IV; C1: III **→** I, C2: III **→** II, C3: IV **→** II, C4: IV **→** I. I belts are present in both EcoRI/Hpa II and EcoRI/Msp I digestion combination, II belts are present only in EcoRI/Hpa II, and III belts are present only in EcoRI/MspI*.

Collectively, the band numbers of demethylation in root and leaf under 5-azaC treatment were more than those in methylation, suggesting that DNA demethylation was induced significantly by the application of 5-azaC. Moreover, the demethylation degree was more significant under 5-azaC treatment in dose-dependent manner. In addition, it was also shown that in root and leaf, the band patterns for the increase in methylation were almost II → IV, and the band patterns for the increase in demethylation were mainly IV → II.

## Discussion

*R. glutinosa* is a perennial herb and is one of the traditional Chinese medicines (Chen et al., 2021). It is well known that iridoid glycoside, which belongs terpenoids, has a wide range of pharmacological effects and important economic value (Keeling and Bohlmann, 2006; Bohlmann and Zerbe, 2012). The previous study confirmed that most prenyltransferases involved in terpenoid backbone biosynthesis were the member of FPP/GGPP synthase families, and all prenyltransferases were interacted with biotin carboxylase CAC2 (Chen et al., 2021). A reference genome of *R. glutinosa* was reported using Nanopore technology, Illumina, and Hi-C sequencing, in which the expansion of the UDP-dependent glycosyltransferases and the terpene synthase gene families was demonstrated (Ma et al., 2021). Nevertheless, the regulation of genes involved in iridoid glycoside biosynthesis was rarely reported in *R. glutinosa*.

In our study, 357 unigenes involved in iridoid glycoside biosynthesis were identified and annotated (Supplementary Table 4). GO enrichment indicated that these unigenes above were located in the ample organelle (Figure 1B), suggesting that they were widely distributed in cell. In current, the study of secondary metabolism in plants is mainly focused on the key synthetic enzymes, such as *DXS, DXR, GPPS, G10H*, and *10HGO*. Herein, these genes were cloned from *R. glutinosa*, and the further analysis showed that the intron was only existed in *DXS* (Supplementary Figure 5). DXS, DXR, GPPS, G10H, and 10HGO were predicted exist in cytoplasm and chloroplast (Supplementary Table 6). The co-occurrence ratios of *DXS, DXR*, or *GPPS* were higher than those in *G10H* and *10HGO* in plant, and they were conservative in Viridiplantae (Figure 3, Table 1). Subsequently, the expression level of *DXS, DXR, GPPS, G10H*, and *10HGO* was detected by qRT-PCR in root and leaf, respectively (Figure 4). *DXS, DXR, GPPS*, and *10HGO* in root were higher than those in leaf, and the expression levels of *DXS* at I stage and *GPPS* at M stage in leaf were higher than those in root. Analogously, the expression level of *MtDXS2* is abundant in root, but low in tissues above-ground in *M. truncatula* (Zhou W. et al., 2016). Unexpectedly, the expression level of *G10H* in leaf was above 3-fold change compared with root (Figures 4A,B). Similarly, *SmG10H* transcripts were much more abundant in the leaf than in either the root or the stem of *Swertia mussotii* (Wang et al., 2010).

DNA methylation could affect plant growth and development (Candaele et al., 2014; Yamamuro et al., 2014; Zhou W. et al., 2016), SMs accumulation, and the expression of synthetase gene associated with SMs (Qi et al., 2016; Wang B. et al., 2018; Wang et al., 2019). Studies have shown that the gene expression of the secondary metabolic biosynthesis pathway was upregulated under 5-azaC (a DNA methylation inhibitor) treatment (Ni et al., 2014; Jiang et al., 2018; Li et al., 2018; Bacova et al., 2019; Luo et al., 2021). In our research, under 15 and 50 μM 5-azaC treatment, the expression of *DXS, DXR, GPPS, G10H*, and *10HGO* was upregulated in root and leaf, especially 50 μM (Figure 5). In addition, the accumulation of secondary metabolites was also increased under 5-azaC treatment (Jiang et al., 2018; Li et al., 2018; Bacova et al., 2019; Luo et al., 2021). Ample studies revealed that the expression level of *DXS, DXR, GPPS, G10H*, and *10HGO* could affect the accumulation of the corresponding secondary metabolites. For example, the increase in carotenoid and vitamin E content was observed, when DXS was overexpressed (Estevez et al., 2001). By contrast, the content of artemisinin decreased significantly in *Artemisia annua* with *DXR* no-expression (Wang C. et al., 2018). Overexpression of geranyl diphosphate synthase small subunit 1 (*LcGPPS.SSU1*) could enhance the monoterpene production in *L. cubeba* and tobacco (Zhao et al., 2019). *G10H* overexpression increased significantly the accumulation of strictosidine, vindoline, and catharanthine (Pan et al., 2012). Taken together, the iridoid glycoside accumulation was detected under 5-azaC treatment. It was found that the iridoid glycoside accumulation was also increased obviously under 15 and 50 μM 5-azaC treatment in root, especially 50 μM 5-azaC (Figure 6A). Iridoid glycoside content in root was higher than those in leaf under control and 5-azaC treatment group (Figures 6A,B). It was worth noting that under 50 and 100 μM 5-azaC treatment, the iridoid glycoside content in root was above 2-fold than those in leaf at M stage (Figures 6A,B). Similarly, ganoderic acid (GAs) accumulation was promoted by 5-azaC in *Ganoderma lucidum*, and the key enzyme genes (*hmgr, sqs, se*, and *ls*) of GAs biosynthesis expression level were upregulated (Lan, 2016). The *SmDMLs* (DEMETER-like DNA glycosylases) expression was downregulated by 5-azaC treatment in *Salvia miltiorrhiza*, in which the tanshinone content was increased significantly (Jiang et al., 2018; Li et al., 2018), suggesting that the tanshinone accumulation was pronounced by 5-azaC induced DNA demethylation. The effect of 5-azaC on secondary metabolites accumulation and genes expression was also confirmed in *Chlamydomonas reinhardtii* (Bacova et al., 2019), *Dendrobium* (Ni et al., 2014), and *Pogostemon cablin* (Luo et al., 2021). In summary, we speculated that the upregulation of *DXS, DXR, GPPS, G10H*, and *10HGO* induced by 5-azaC treatment was involved in the iridoid glycoside accumulation in *R. glutinosa*.

Previous studies confirmed that the gene expression level was increased by applicating 5-azaC, in which the DNA demethylation was induced (Jiang et al., 2018; Li et al., 2018; Zhu, 2020). Our study showed that at M stage of *R. glutinosa*, the total and full methylation ratios under 15 and 50 μM 5-azaC treatment were higher compared with control, but decreased significantly under 100 μM 5-azaC (Figure 7A). The hemi-methylation ratio was higher than other groups under 15 μM 5-azaC treatment (Figure 7A). Compared with leaf, the total and full methylation levels of root were lower at I and M stage (Figures 7B,C). Genome DNA demethylation levels could be presented by 5mC content. In potato, the 5mC levels were reduced by 5-azaC treatment (Law and Suttle, 2005). 5mC content and total methyltransferase activity were decreased, when treated by 5-azaC in peony (Zhang et al., 2020). Our results showed that 5mC contents in root and leaf were decreased significantly under 5-azaC treatment in dose-dependent manner (Figures 7D,E). In addition, the internal reason for the methylation changed was also revealed in this study. The methylation increasing band patterns were almost II → IV, and demethylation was mainly IV → II (Table 2). Moreover, the methylation changes in the leaf were more active than those in root. In addition, 5mC content was decreased under 15 and 50 μM 5-azaC, but full methylation level showed an increasing trend in root (Figures 7A,D). We speculated that CCGG methylation site could be detected by MSAP, but ^m^C^m^CGG/GGC^m^C^m^ sites could not be detected. Thus, the increase or decrease in DNA methylation could not be determined due to the limitation of MSAP, such as I → II, II → I, III → IV, or IV → III. These methylation sites might transform into full methylation (band type III) or hemi-methylation sites (band type II), followed by the methylation ratio which was higher than those of control.

At present, the study showed that the change trend of DNA methylation was dynamically related to isoflavone synthesis in *Robinia pseudoacacia*, and the isoflavone synthesis was blocked by 5-azaC treatment (Xia, 2006). Overexpressing demethylase gene *ROSI* in transgenic tobacco could promote significantly the gene expression of flavonoid metabolism (Bharti et al., 2015). Moreover, the upregulation of the expression of genes involved in flavonoid synthesis was induced, when the demethylase gene was overexpressed in transgenic *hybrid poplars*, followed by increasing the flavonoid accumulation in apical meristem, scale bud, and apical bud (Daniel et al., 2017). Ample studies indicated that DNA demethylation was induced by 5-azaC treatment, which increased the expression of key enzyme genes in secondary metabolic pathway, resulting in the secondary metabolite accumulation.

Overall, our results suggested that under 5-azaC treatment, the demethylation of iridoid glycoside synthetase genes in *R. glutinosa* might activate the expression of these genes, followed by inducing the iridoid glycoside accumulation. However, the terpenoid synthesis pathway is very complex, and the mechanisms of regulation of DNA methylation on iridoid glycoside synthesis were unclear. Thus, the roles of epigenetic regulation in iridoid glycoside synthesis were still investigated in near future.

## Data Availability Statement

The datasets used and analyzed in the current study are available from the corresponding author on reasonable request.

## Author Contributions

TD and HD conceptualized the study. JS, TD, and YL designed the methodology. TD, SS, YW, RY, and XD were involved in formal analysis. TD, SS, and YW wrote—original draft. JS, PC, and HD were involved in writing—review and editing. HD was involved in funding acquisition. All authors have read and agreed to the published version of the manuscript.

## Funding

This research was completed under the financial aid of the National Science Foundation of China (Nos. 31870312 and 31500262), Fund of Henan Normal University (No. 2019JQ01), and the Key Project of Henan Province (No. 212102110405) in China.

## Conflict of Interest

The authors declare that the research was conducted in the absence of any commercial or financial relationships that could be construed as a potential conflict of interest.

## Publisher's Note

All claims expressed in this article are solely those of the authors and do not necessarily represent those of their affiliated organizations, or those of the publisher, the editors and the reviewers. Any product that may be evaluated in this article, or claim that may be made by its manufacturer, is not guaranteed or endorsed by the publisher.

## Abbreviations

10HGO: 8-hydroxygeraniol dehydrogenase
5-azaC: 5-azacytidine
5mC: 5-methyl cytosine
DMAPP: dimethylallyl diphosphate
DXR: 1-deoxy-D-xylulose-5-phosphate reductase
DXS: 1-deoxyxylulose-5-phosphate synthetase
G10H: geraniol 10-hydroxylase
GPP: geranyl diphosphate
GPPS: geranyl pyrophosphate synthase
HPLC: high-performance liquid chromatography
IPP: isoprene diphosphate
MEP: 2-C-methyl-D-erythritol-4-phosphoric acid pathway
MSAP: methylation-sensitive amplified polymorphism
qRT-PCR: quantitative real-time PCR
SMs: secondary metabolites.
